# Mesh Ti6Al4V Material Manufactured by Selective Laser Melting (SLM) as a Promising Intervertebral Fusion Cage

**DOI:** 10.3390/ijms23073985

**Published:** 2022-04-03

**Authors:** Agata Przekora, Paulina Kazimierczak, Michal Wojcik, Emil Chodorski, Jacek Kropiwnicki

**Affiliations:** 1 Independent Unit of Tissue Engineering and Regenerative Medicine, Medical University of Lublin, Chodzki 1 Street, 20-093 Lublin, Poland; paulina.kazimierczak@umlub.pl (P.K.); michal.wojcik@umlub.pl (M.W.); 2 ChM sp. z o.o., Lewickie 3b Street, 16-061 Juchnowiec Kościelny, Poland; emil.chodorski@chm.eu (E.C.); jacek.kropiwnicki@chm.eu (J.K.)

**Keywords:** biomaterial, spinal implant, metallic implants, polyether-ether-ketone (PEEK), load-induced static subsidence test, biocompatibility, osteoconductivity, osteogenic differentiation

## Abstract

Intervertebral cages made of Ti6Al4V alloy show excellent osteoconductivity, but also higher stiffness, compared to commonly used polyether-ether-ketone (PEEK) materials, that may lead to a stress-shielding effect and implant subsidence. In this study, a metallic intervertebral fusion cage, with improved mechanical behavior, was manufactured by the introduction of a three-dimensional (3D) mesh structure to Ti6Al4V material, using an additive manufacturing method. Then, the mechanical and biological properties of the following were compared: (1) PEEK, with a solid structure, (2) 3D-printed Ti6Al4V, with a solid structure, and (3) 3D-printed Ti6Al4V, with a mesh structure. A load-induced subsidence test demonstrated that the 3D-printed mesh Ti6Al4V cage had significantly lower tendency (by 15%) to subside compared to the PEEK implant. Biological assessment of the samples proved that all tested materials were biocompatible. However, both titanium samples (solid and mesh) were characterized by significantly higher bioactivity, osteoconductivity, and mineralization ability, compared to PEEK. Moreover, osteoblasts revealed stronger adhesion to the surface of the Ti6Al4V samples compared to PEEK material. Thus, it was clearly shown that the 3D-printed mesh Ti6Al4V cage possesses all the features for optimal spinal implant, since it carries low risk of implant subsidence and provides good osseointegration at the bone-implant interface.

## 1. Introduction

Spinal implants for clinical applications should reveal some key features, like resistance to microbial colonization, high biocompatibility and bioactivity (both allowing for good implant osseointegration with host bone), appropriate mechanical parameters tailored to the intended use of the implant in a biomechanical context (e.g., sufficient compressive strength and low implant subsidence tendency), and favorable medical imaging properties (reduced artifacts) [1]. Clinical success of load-bearing spinal implants (such as intervertebral fusion cages) depends highly on the osseointegration process, which is defined as the formation of a direct connection between the orthopedic implant and the surrounding host bone, without intervention of connective tissue at the bone-implant interface [2,3]. Thus, it is not surprising that good adhesion and proliferation of osteoblasts/mesenchymal stem cells on the surface of the spinal implants are among the most important requirements of orthopedic materials.

To meet all the above-mentioned criteria, load-bearing medical implants for spine surgery are frequently produced using the following materials that have sufficient mechanical strength: stainless steel, pure titanium and its alloys (e.g., titanium-aluminum-vanadium, Ti6Al4V, nickel-titanium, or nitinol), cobalt-chromium, tantalum, and polyether-ether-ketone (PEEK) [1]. Ti6Al4V and PEEK are the ones most often used for the fabrication of spinal implants [4].

PEEK, which is a semi-crystalline thermoplastic, was introduced into clinical applications in the early 1990s. Compared to metallic implants, PEEK has superior mechanical parameters, since it reveals reduced Young’s modulus, similar to human bone [5,6]. Nevertheless, conventional PEEK implants have not only a smooth surface, due to their production process, which involves injection molding or machining, but also PEEK implants have hydrophobic character. Both of these features significantly hinder bonding of PEEK implants to bone tissue. Therefore, PEEK implants are generally characterized by poor osseointegration, that may lead to surgery failure [7,8,9,10].

Spinal implants made of Ti6Al4V alloy show excellent biocompatibility and osteoconductivity, since they allow for good osteoblast adhesion and proliferation. However, Ti6Al4V alloy reveals a higher Young’s modulus compared to human bone, that may lead to bone atrophy, followed by implant loosening, due to the stress-shielding effect [11,12], or cage subsidence, due to penetration of the implant into the inferior vertebra, causing reduction in height of the fused segment [13]. Therefore, Ti6Al4V implants exhibit superior osteoconductivity but inferior mechanical parameters, compared to PEEK materials. Nevertheless, by reducing the Young’s modulus of Ti6Al4V alloy it is potentially possible to achieve an ideal intervertebral fusion cage, with optimal stiffness, low implant subsidence tendency, and high biocompatibility.

One of the methods that may be used to reduce the Young’s modulus of metallic implants involves introduction of the appropriate porosity into their structure [11,14,15]. Importantly, introduction of porosity to metallic implants will not only reduce their Young’s modulus, but also improve their osseointegration, since it is well known that rough and porous surfaces enhance cell adhesion and facilitate bone ingrowth deep into the implant [12,16,17,18,19,20,21]. According to available literature, the most optimal osseointegration is observed for orthopedic implants with macroporosity of at least 60%, and pore sizes in the range of 200–1200 μm [18,22].

Appropriate porosity of metallic implants may be achieved by application of additive manufacturing (AM) technology, also known as three-dimensional (3D) printing, and rapid prototyping [23]. AM is used in industrial production of spinal implants to primarily meet the requirements for a low implant subsidence tendency, and improved vascularization and osseointegration. For the production of metallic implants, various AM approaches may be used: including, powder bed fusion (PBF), direct energy deposition (DED), laminated object manufacturing (LOM), selective laser sintering (SLS), and selective laser melting (SLM) [24]. The last-mentioned technique, SLM, deserves special attention, since it is frequently used for the fabrication of spinal implants made of titanium and its alloys, stainless steel, and cobalt-chromium [25]. The SLM technique involves selective sintering of metallic powders applied layer by layer with a laser beam, until the final product is obtained.

**Figure 1:**
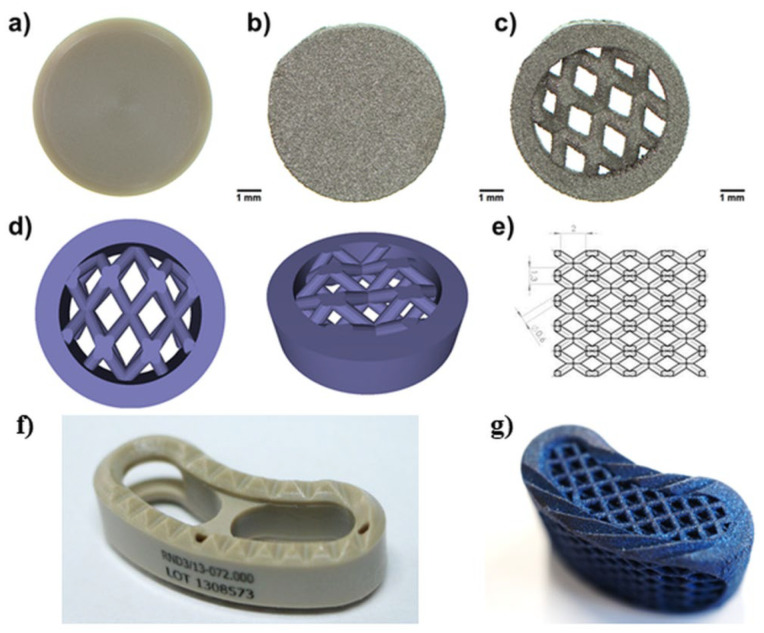
Images presenting tested materials: (**a**) solid PEEK, (**b**) 3D-printed solid Ti6Al4V, (**c**) 3D printed mesh Ti6Al4V, (**d**) the 3D model for the production of the mesh sample was designed using SOLIDWORKS^®^ software, (**e**) dimensions of the mesh part of the sample model, (**f**) TLIF PEEK intervertebral cage, (**g**) mesh TLIF 3D-Ti intervertebral cage (made of Ti6Al4V).

The aim of this research was to apply the SLM technique to the production of porous 3D-printed Ti6Al4V material, with reduced stiffness and a mesh structure analogous to that used in 3D-printed spinal implants, having the ability to promote osseointegration. In this study, properties of three different samples were compared: (1) PEEK, with a solid structure prepared by the machining process, (2) 3D-printed Ti6Al4V, with a solid structure, and (3) 3D-printed Ti6Al4V, with a mesh structure (Figure 1a–e. Mechanical behavior of the samples was characterized by means of a static compression test (maximum compression load was estimated) and a load-induced static subsidence test, which allowed determination of the tendency of the implants (Figure 1f, 1g) to subside into polyurethane foam, mimicking the bone of vertebral bodies. Moreover, biological properties (biocompatibility, osteoconductivity, bioactivity) of the samples were assessed in vitro, using osteoblast cell lines and bone-marrow derived mesenchymal stem cells.

It should be noted that both PEEK and Ti6Al4V are frequently used in industrial production of intervertebral fusion cages. Thus, the comprehensive comparative studies performed not only allowed us to gain valuable knowledge as to which material is more desirable for clinical applications, but also answered the question as to whether introduction of mesh porosity into Ti6Al4V material influences its biological properties. 

## 2. Results and Discussion

### 2.1. Mechanical Test

Spinal implants consist of load-bearing materials exposed to high mechanical loads. Thus, they should be characterized by appropriate mechanical parameters, to ensure optimal support for new bone formation. For this reason, intervertebral cages are frequently made of metallic materials. Nevertheless, metallic implants reveal significantly greater Young’s modulus, compared to human bone. Thus, their implantation is often associated with a high risk of cage subsidence or the stress-shielding effect causing implant loosening [11,12]. Within this study, the SLM-manufacturing technique was applied to introduce mesh porosity into Ti6Al4V samples to reduce stiffness and lower the risk of cage subsidence after implantation. The tested samples were assessed for their ability to withstand expected physiological static loads, and were checked as to whether manufactured 3D-printed mesh Ti6Al4V intervertebral cages meet design requirements and are safe for use in accordance with the intended purpose.

#### 2.1.1. Static Compression Test

**Figure 2:**
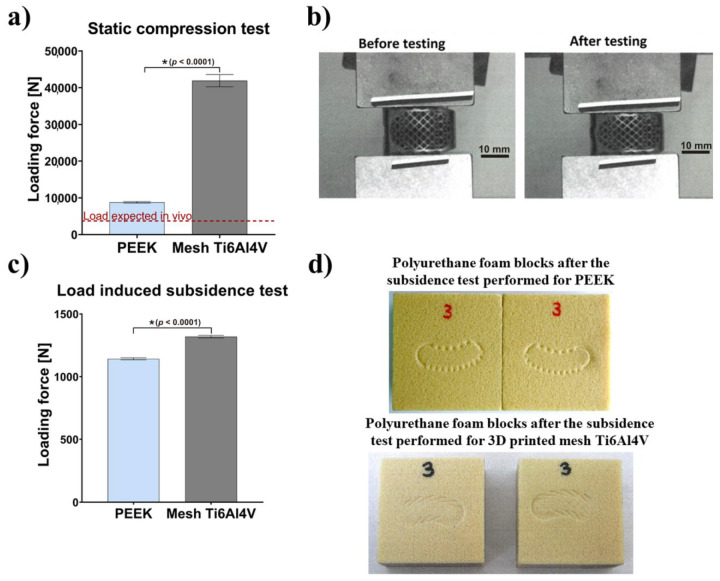
Mechanical behavior of tested intervertebral cages: (**a**) Results obtained with a static compression test in relation to the in vivo expected load (* statistically different results compared to PEEK cage according to unpaired *t*-test); (**b**) Images presenting Ti6Al4V intervertebral cage before and after static compression testing; (**c**) Results obtained with a load-induced subsidence test (* statistically different results compared to PEEK cage according to unpaired t-test; (**d**) Images presenting the appearance of the polyurethane foam blocks after the subsidence test performed for PEEK cage and 3D-printed mesh Ti6Al4V cage.

The results comparing mechanical behavior of Ti6Al4V and PEEK implants, in relation to expected load in vivo, are presented in Figure 2a. The failure mode for PEEK samples was plastic deformation. For 1 mm reduction of the sample, average load of 8779 ± 236 N was achieved. After load removal, the sample returned to a shape similar to the original. Plastic deformation was recorded with an average value of 0.86 mm. The failure mode for mesh Ti6Al4V cages was sample breakage. For 1 mm reduction of the sample, a mean load of 41,915 ± 1664 N was achieved. After load removal, the sample did not return to the original shape. The titanium specimen before and after the static compression test is shown in Figure 2b. The load recorded for 1 mm PEEK sample reduction was more than 2.5-fold greater than the load expected in vivo (about 3400 N). According to test results reported in the available literature, compression force that is destructive for vertebral body is in the range of 4971 N to 8572 N [26]. Visual inspection and measurement of the PEEK samples after the test showed only small (0.86 mm) plastic deformation of teeth and no structural damage to the implant body. The load recorded for 1 mm mesh Ti6Al4V implant (41915 N) was more than 12-fold greater than the expected load in vivo resulting from usual human physical activity, including jumps and lifting [26], and over 4.8-fold greater than the compression force that is destructive for a vertebral body.

Compressive strength recorded for both types of implants (PEEK and Ti6Al4V) was significantly higher than the expected load in vivo and, therefore, may be considered as sufficient. Importantly, the Ti6Al4V cage showed significantly superior mechanical stability and resistance to extremely high mechanical loads, compared to the PEEK cage. Nevertheless, it should be noted that in normal circumstances, when the healing process goes well, the implant is subjected to high loads only in the initial period of the treatment. After the osseointegration process and bone remodeling, the implant is overgrown by bone and becomes a single cohesive block of two adjacent vertebrae. Then, the loads are transferred via bone tissue and adjacent structures.

#### 2.1.2. Load-Induced Subsidence Test

Test results showed that mean load recorded at 3 mm subsidence was equal to 1142 ± 8 N for the PEEK implant and 1319 ± 10 N for the mesh Ti6Al4V cage. There was no visible damage to the tested implants (Figure 2c). Figure 2d shows polyurethane foam blocks, which reflect implant subsidence in human cancellous bone. Importantly, the average load value recorded for the mesh Ti6Al4V implant was 15.5% higher than for the PEEK cage. Thus, the tendency of the mesh Ti6Al4V intervertebral cage to subside into the polyurethane foam (simulating the vertebral bone) is about 15% lower, compared to the PEEK cage. Taking into account the nature of the load (compression), design of the implant and a large safety factor (2–12), in relation to expected in vivo loads (3400 N), it was decided not to perform fatigue tests as they would not provide any useful information. It should be noted that application of loads higher than physiological (over 3400 N) i.e., loads close to the destructive force for the human vertebral body, has no rational basis.

It should be noted that level of subsidence of the intervertebral fusion cage is mostly impacted by stress distribution at the implant-bone contact area, which results from the structure of the implant. Porous structure may be created in titanium implants, by using additive manufacturing methods. However, the machining processes used to produce PEEK implants cannot provide high levels of implant porosity. Importantly, a study by Lim et al. showed that subsidence likelihood for a non-porous intervertebral fusion cage was 2.33 times higher in flexion motion than for a porous cage, 1.16 times higher in extension motion, 1.9 times higher in axial rotation motion and 1.81 times higher in lateral bending motion [27]. Moreover, Krafft et al. demonstrated that porous structure led to maximized bone-implant contact area and minimized stress-shielding effect [28]. Results obtained by Chatham et al. proved also that a larger implant-to-bone contact interface provided more even stress distribution, which lowered the risk of subsidence [29]. Furthermore, in a finite element model proposed by Liu et al., stress tended to be more concentrated at the periphery of endplates [30]. In turn, Wang et al. analysed optimized porous structures of spinal implants, which provided both even stress distribution and desired stiffness. The studies revealed that maximal stress at the endplates for a non-porous fusion cage was higher for all loading conditions used in the study [31]. Therefore, it was suggested that introduction of a mesh porous structure and appropriate surface topography to the Ti6Al4V implant leads to decreased stress, which results in lower subsidence in the case of a porous titanium fusion cage, in comparison to a PEEK cage. 

### 2.2. Wettability Test

It is well known that adhesion of osteoblasts and mesenchymal stem cells to bone implants is primarily mediated by adsorption of various adhesive proteins. Importantly, adsorption of these proteins depends highly on the following physicochemical, and microstructural, properties of the implant surface: chemistry, wettability, microporosity, or roughness. Surface wettability has been identified as one of the most important factors in regulating the effectiveness of cell adhesion to implants [32,33]. It is worth noting that poor wettability (hydrophobicity) of PEEK material is considered the main factor responsible for its inferior osseointegration [7,8]. 

**Figure 3:**
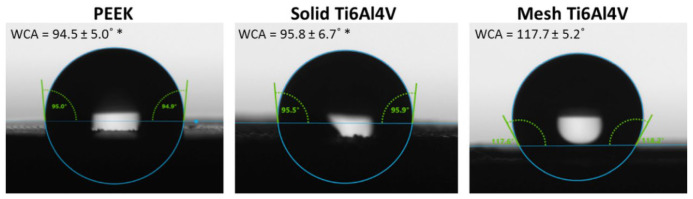
Water contact angle measurements performed for tested materials (* statistically different results compared to mesh Ti6Al4V sample, according to One-way ANOVA followed by Tukey’s multiple comparison test).

The wettability test we performed confirmed the hydrophobic character of PEEK, since the water contact angle (WCA) was equal to 94.5°. Surprisingly, solid titanium samples showed similar WCA to PEEK, whereas introduction of mesh porosity significantly increased WCA to 117.7° (Figure 3). Although mesh and solid Ti6Al4V samples had the same chemical composition, the applied printing conditions might have affected topography of the resultant samples, and thus their wettability. For instance, during the printing process the mesh sample was exposed to laser for a longer period of time. Moreover, surface of the mesh Ti6Al4V material was characterized by the presence of a greater amount of unmelted titanium particles, that could have hindered spreading of the water droplets during the test.

### 2.3. Bioactivity Test

Bioactivity of spinal implants, which is defined as their ability to form a carbonated hydroxyapatite layer on their surfaces after implantation or immersion in body fluid, is a very important feature, since it provides for better bonding to bone tissue and improved osseointegration [34,35]. It is known that rough surfaces of biomaterials induce apatite precipitation and provide improved bioactivity of the material [18,36,37].

**Figure 4:**
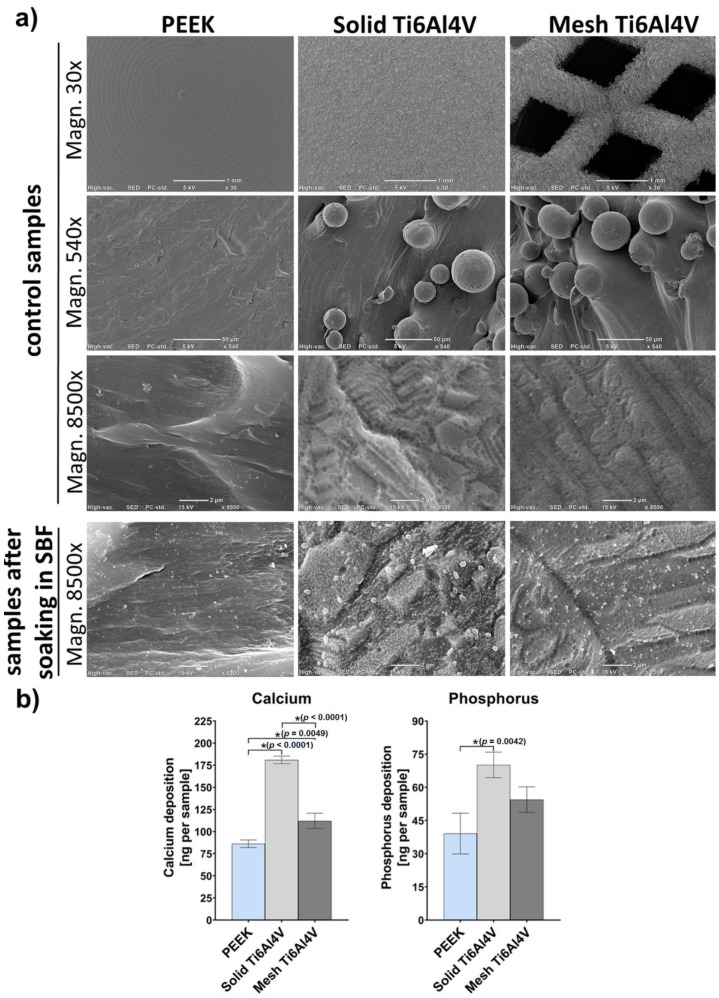
(**a**) Microstructure of samples visualized by SEM before (control samples) and after soaking in SBF for 28 days. (**b**) Quantitative evaluation of the calcium and phosphorus deposition on the surfaces of the samples after soaking in SBF (* indicates statistically significant results according to One-way ANOVA followed by Tukey’s multiple comparison test).

Scanning electron microscope (SEM) micrographs of untreated control samples (without incubation in SBF) clearly showed high surface roughness of both 3D-printed Ti6Al4V samples (solid and mesh) (Figure 4a). In contrast, the PEEK sample exhibited a rather smooth surface. The only roughness observed on the PEEK disc resulted from its production process, and primarily consisted of parallel longitudinal grooves and ridges. Immersion of the samples in SBF clearly demonstrated that the solid Ti6Al4V sample showed the highest bioactivity (apatite-forming ability) among all tested materials. Solid titanium was covered by many precipitates of calcium phosphates, whereas PEEK exhibited the lowest apatite-forming ability. SEM observation was confirmed by quantitative analysis of calcium and phosphorous deposition on the surface of the samples (Figure 4b), which clearly showed the greatest amount on the solid Ti6Al4V, compared to mesh titanium and PEEK. Observed poor bioactivity of PEEK is consistent with the results obtained by other authors and most likely is due to its smooth surface. Reduced bioactivity of the mesh sample may be explained by its higher hydrophobicity, compared to the solid titanium material.

### 2.4. Cell Culture Experiments

#### 2.4.1. Osteoblast Viability, Adhesion and Growth

Spinal implants should provide good osseointegration with the host tissue. To ensure optimal bonding to the bone, the implants must reveal high osteoconductivity, by being favorable for osteoblast adhesion, proliferation, and differentiation [3,38,39]. It was suggested that biomaterials exhibiting good bioactivity, biocompatibility, and osteoconductivity should provide good osseointegration at the implantation site in vivo.

**Figure 5:**
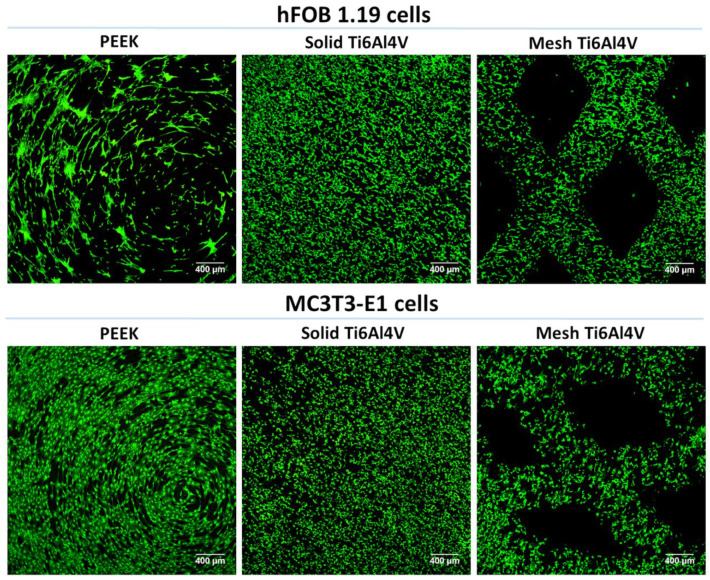
CLSM images presenting viability of osteoblasts cultured for 48 h on the surface of tested materials (green fluorescence—viable cells stained with calcein-AM; magnification 40×, scale bar = 400 µm).

Within this study, osteoblast viability, adhesion, and growth on the tested implants were assessed using two in vitro cellular models: (1) human fetal osteoblasts (hFOB 1.19 cell line) and (2) mouse calvarial preosteoblasts (MC3T3-E1 Subclone 4 cell line). We performed live/dead staining of the cells cultured on the samples, which showed that all materials were non-toxic and favored cell adhesion (Figure 5). The cells were viable and emitted only green fluorescence. No dead cells (red fluorescence) were detected. Interestingly, confocal laser scanning microscope (CLSM) images revealed that human osteoblasts (hFOB 1.19) cultured on the PEEK sample had a tendency to form clusters. Moreover, noticeably fewer hFOB 1.19 cells were observed on PEEK. In the case of MC3T3-E1 preosteoblasts, there were no differences in osteoblast growth between tested samples.

**Figure 6:**
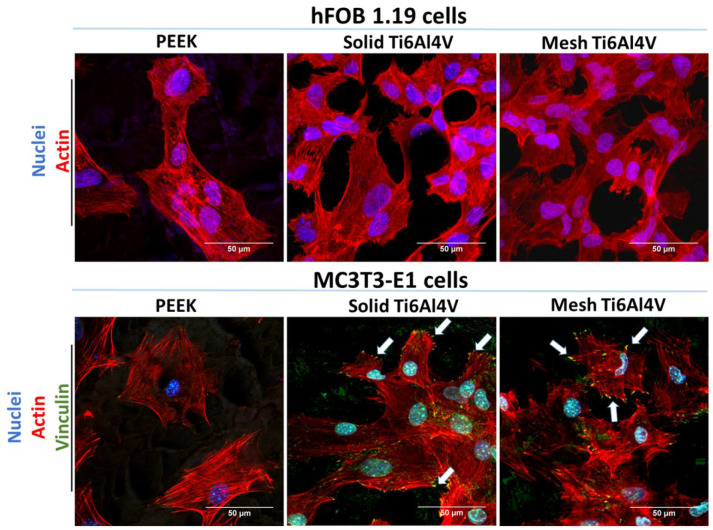
CLSM images showing osteoblasts’ morphology and adhesion after 48-h culture on the tested samples; hFOB 1.19 cells: red fluorescence—F-actin, blue fluorescence—nuclei; MC3T3-E1 cells: green fluorescence—vinculin, red fluorescence—F-actin, blue fluorescence—nuclei (white arrows indicate FAPs visible as green dot-like structures on the edges of the cells); magnification 400× + 2f, scale bar = 50 µm.

Analysis of cell morphology after fluorescent staining of the cytoskeleton confirmed inferior adhesion of human osteoblasts to PEEK, since high magnification CLSM images revealed better spreading of hFOB 1.19 cells on both Ti6Al4V samples, compared to the non-metallic sample (Figure 6). In the case of MC3T3-E1 preosteoblasts, the cells showed similar spreading and morphology on all tested materials. However, cells on PEEK were vinculin-negative and did not form focal adhesion plaques (FAPs). Vinculin is a protein involved in the linking of integrin to F-actin, forming FAPs that provide mechanical linkages to extracellular matrix (ECM) as well as implant surface covered by adsorbed proteins [40,41]. Unlike cells on PEEK, MC3T3-E1 preosteoblasts grown on both 3D-printed Ti6Al4V materials formed typical FAPs, proving their strong adhesion to the titanium samples. 

**Figure 7:**
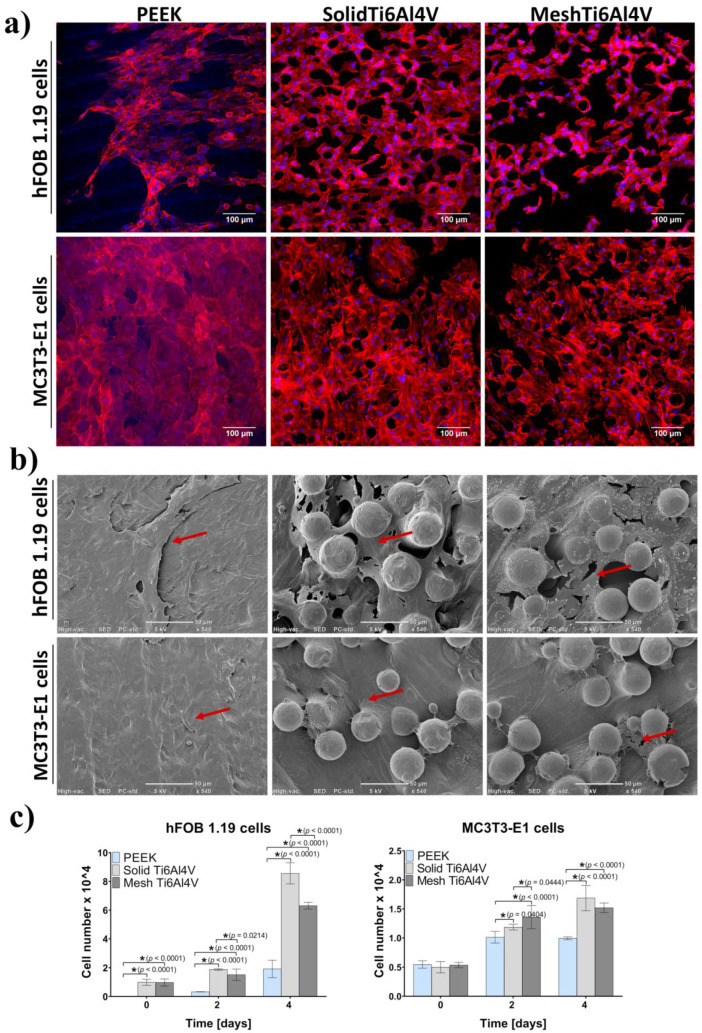
Cell growth and proliferation on the tested materials: (**a**) CLSM images presenting osteoblasts’ growth after four-day culture (red fluorescence—actin cytoskeleton, blue fluorescence—nuclei; magnification 200×, scale bar = 100 µm); (**b**) SEM micrographs presenting osteoblasts growth after four-day culture (red arrows indicate sheet of osteoblasts on the samples; magnification 540×, scale bar = 50 µm), (**c**) quantitative evaluation of cell proliferation (* indicates statistically significant results according to One-way ANOVA followed by Tukey’s multiple comparison test).

Better growth and proliferation of hFOB 1.19 osteoblasts on the surface of both Ti6Al4V materials, compared to PEEK, was also proven by visualization of the samples after 4-day culture using CLSM and SEM. There were noticeably fewer hFOB 1.19 cells on the surface of PEEK compared to both titanium materials (Figure 7a,b). Moreover, quantitative evaluation of cell proliferation revealed that there were 4-fold and 3-fold more hFOB 1.19 cells on the surface of solid Ti6Al4V and mesh Ti6Al4V samples, respectively, compared to PEEK (Figure 7c). Interestingly, on the second and fourth day of the culture a significantly greater number of cells was detected on the surface of the solid Ti6Al4V material, compared to the mesh one; indicating that the solid titanium sample was more favorable to proliferation of human osteoblasts. Unlike hFOB 1.19 cells, CLSM and SEM images showed similar growth of MC3T3-E1 preosteoblasts on all tested materials (Figure 7a,b). However, a quantitative assay clearly demonstrated that MC3T3-E1 cells revealed better proliferation on both Ti6Al4V samples, compared to PEEK (Figure 7c). 

According to the available literature, PEEK material does not support cell adhesion and proliferation, most likely due to its hydrophobic character and smooth surface [7,8,9,10]. In this study, both hydrophobic character (Figure 3) and smooth surface of the PEEK sample (Figure 4) were confirmed. Consequently, cell culture tests revealed inferior osteoblast adhesion and proliferation on the PEEK material, compared to both Ti6Al4V samples (although they were also characterized by hydrophobicity), which is consistent with the results presented by other authors. Better osteoblast growth on the metallic samples most likely resulted from higher surface roughness of the titanium materials. Nevertheless, the PEEK sample still allowed for relatively good osteoblast adhesion and growth, so its inferior osteoconductivity, compared to the titanium samples, should not exclude this material from clinical use.

#### 2.4.2. Osteogenic Differentiation of Osteoblasts and Stem Cells

Good osseointegration of spinal implants highly depends on the process of new bone formation and ECM mineralization. Production of the mineralized bone ECM is mediated by osteoblasts and mesenchymal stem cells, which are the source of osteoprogenitor cells at the implantation site [42]. New bone is formed as a consequence of osteogenic differentiation, a complex process divided into three steps: (1) cell proliferation (increased production of fibronectin, moderate synthesis of type I collagen), (2) ECM synthesis (increased production of type I collagen, moderate synthesis of osteocalcin and bone sialoprotein), and (3) ECM mineralization (increased production of osteocalcin and bone sialoprotein, mineral deposition) [42,43,44,45].

**Figure 8:**
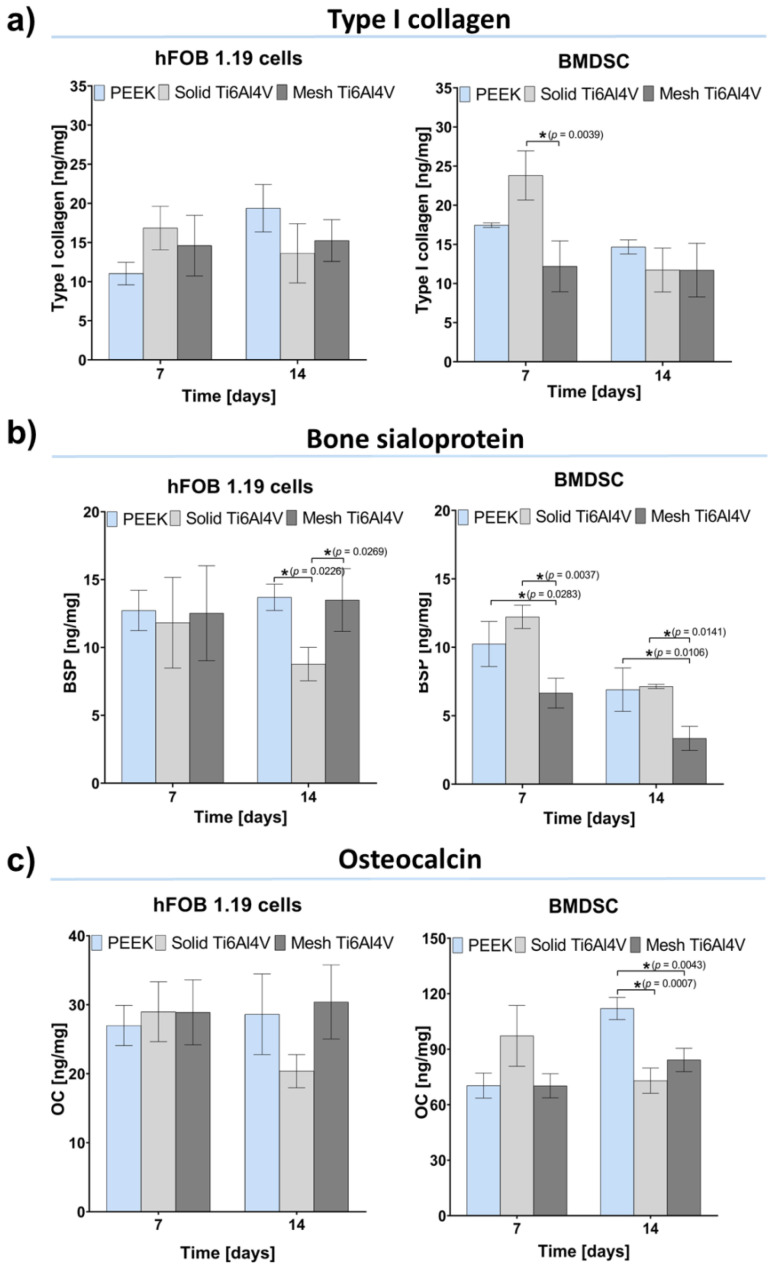
Osteogenic differentiation of the tested materials determined by quantitative evaluation of typical bone formation markers: (**a**) type I collagen (Col I); (**b**) bone sialoprotein (BSP), (**c**) osteocalcin (OC); (* indicates statistically significant results according to One-way ANOVA followed by Tukey’s multiple comparison test).

Within this study, osteogenic differentiation in response to the tested materials was compared using human osteoblasts (hFOB 1.19 cell line) and bone marrow-derived mesenchymal stem cells (BMDSCs). To determine the course of the osteogenic differentiation process on the samples, three main proteins of bone ECM were quantitatively assessed: type I collagen (Col I), bone sialoprotein (BSP), and osteocalcin (OC). Moreover, ECM mineralization was evaluated, using a colorimetric method based on Alizarin Red S (ARS) staining. Performed experiments revealed no statistically significant differences in Col I synthesis between tested samples (Figure 8a). Interestingly, human osteoblasts (hFOB 1.19 cells) generally revealed a comparable level of osteogenic markers in response to all tested materials. The only statistically significant difference was detected for the production of BSP by hFOB 1.19 cells grown on the solid Ti6Al4V sample, where the level of BSP was lower compared to PEEK and mesh titanium (Figure 8b). In the case of BMDSCs, significant differences in the level of late osteogenic markers (BSP and OC) were recorded. BMDSCs cultured on mesh Ti6Al4V produced significantly smaller amounts of BSP compared to those on the solid Ti6Al4V material (Figure 8b). Furthermore, on the 14th day stem cells grown on PEEK produced significantly more OC, compared to those on both titanium samples (Figure 8c). It should be noted that both BSP and OC are proteins associated with ECM mineralization and have the ability to bind bone minerals [42,44,45]. Therefore, reduced detection of OC and BSP in BMDSCs grown on the Ti6Al4V samples was most likely associated with enhanced mineralization of these proteins, which hindered antibody–antigen interactions during conducting of ELISAs. This hypothesis found reflection in results obtained with mineralization assay. 

**Figure 9:**
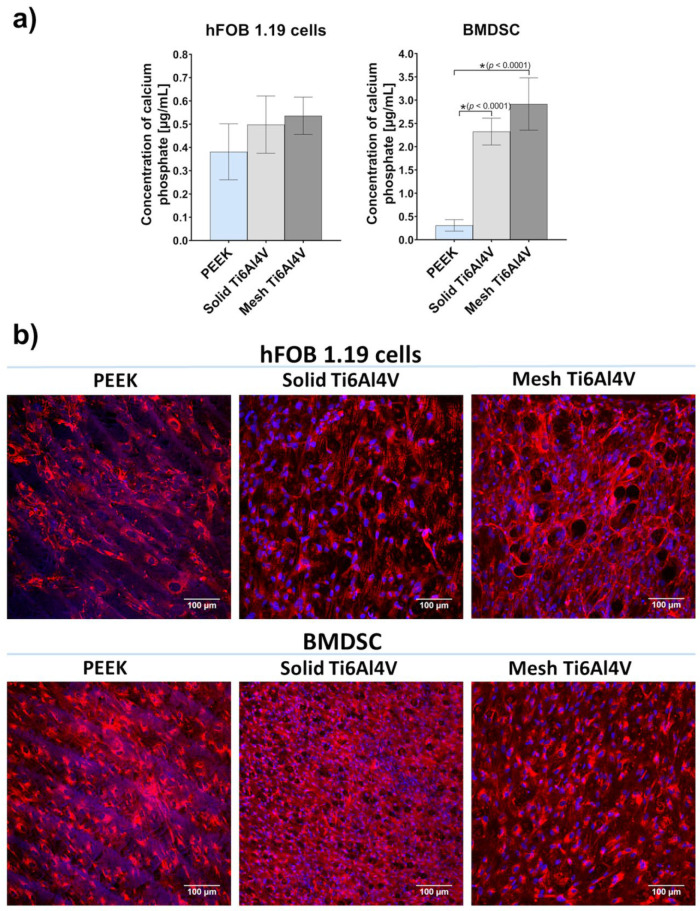
ECM mineralization in response to tested samples: (**a**) quantitative mineral determination based on ARS staining (* indicates statistically significant results according to One-way ANOVA followed by Tukey’s multiple comparison test); (**b**) immunofluorescent staining of Col I fibers acting as a framework for mineral deposition (red fluorescence—Col I, blue fluorescence—nuclei; magnification 200×, scale bar = 100 µm).

The quantitative ARS test showed 4-fold and 7-fold greater (compared to PEEK) mineralization of BMDSCs cultured on the solid Ti6Al4V and mesh Ti6Al4V materials, respectively (Figure 9a). Importantly, stem cells grown on mesh titanium also showed significantly greater mineral deposition, compared to the solid material. Thus, reduced level of BSP for the mesh Ti6Al4V sample, compared to the solid one, may also be explained by significantly enhanced mineralization of stem cells. Although human osteoblasts cultured on the Ti6Al4V materials showed higher levels of mineral compared to PEEK, the results were not statistically significant. It should be noted that ECM calcification highly depends on collagen deposition in the bone ECM, since apatite crystals are formed along the collagen fibers, serving as a framework for mineral deposition [42,44]. Immunofluorescent staining of Col I confirmed the presence of great amounts of this protein on all tested samples seeded with both osteoblasts and stem cells (Figure 9b).

Analysis of the level of bone formation markers in cells grown on the tested samples demonstrated that human osteoblasts had similar osteogenic potential regardless of the growth substrate. Nevertheless, it was clearly proven that both Ti6Al4V materials had the ability to promote the osteogenic differentiation process in stem cells, which exhibited 4-7-fold higher mineralization compared to the cells cultured on PEEK (Figure 9a). Importantly, the highest mineralization level was recorded for the mesh Ti6Al4V sample. It is worth noting that mesh material may also accelerate osseointegration in vivo since it has open porosity, known to support implant vascularization and bone ingrowth. 

## 3. Materials and Methods

### 3.1. Sample Preparation

For all biological experiments and the wettability test, the samples were prepared in the form of discs measuring 8 ± 1.5 mm in diameter and 2 ± 1.0 in height (Figure 1a–c). The PEEK samples (PEEK-OPTIMA^TM^, Invibio Ltd., Rotherham, UK) were produced by mechanical processing. A titanium alloy (Ti6Al4V) destined for the production of implantable medical devices, that conforms to ISO 5832-3 standard, was used for the preparation of 3D-printed samples. The sample solids for 3D printing were designed using SOLIDWORKS^®^ software (Figure 1d,e). SLM-250 Metal 3D Printer (SLM Solutions Group AG, Lübeck, Germany) and Ti6Al4V powder (TLS Technik GmbH, Hartenstein, Germany) were used to make the titanium samples. Printing parameters were optimized by ChM sp. z o.o., based on available reports [46], and are protected by the manufacturer. In general, the parameters were as follows: a layer thickness of 30 μm, laser power of 200 W, working plate temperature of 200 °C.

After printing, the samples were heat treated in an oven at 920 °C for 4 h to eliminate internal stress. Then the supports were removed. All samples were washed twice in an ultrasonic cleaner for 60 min at 37 kHz, and dried for 60 min. Then, the samples were washed and dried in a washer-disinfector according to the implant washing procedure compliant with the ISO 15,883 Standard. The samples were subjected to a steam sterilization process (134 °C, 15 min) that was accompanied by efficiency indicators: chemical indicator (TWINDICATOR, Sterilization Monitor proper; ISO 11140-1:2006, Class 4), biological indicator (EZTest Self-Contained Biological Indicator for Steam Sterilization, MesaLabs, München, Germany), and microbiological indicator (samples were inoculated with the test organism; a suspension of Geobacillus stearothermophilus ATCC 7953 spores). The effectiveness of the sterilization process was confirmed by microbiological testing of the inoculated, and then sterilized, samples.

### 3.2. Mechanical Tests

For mechanical research purposes, the final products of commercially available spinal implants made of PEEK and mesh Ti6Al4V (TLIF PEEK intervertebral cage and TLIF 3D-Ti intervertebral cage, respectively, manufactured by ChM sp. z o.o., Juchnowiec Kościelny, Poland) were used. The cages were fabricated by different methods: (1) TLIF PEEK intervertebral cage, size: 7 *×* 26/0°, produced by mechanical processing (Figure 1f), (2) TLIF 3D-Ti intervertebral cage, size: 16 × 26/0° with a mesh structure, produced by SLM (Figure 1g). The implants were subjected to a static compression test that was performed according to the ASTM F2077-11 and the ASTM F2077-18 standards (Test Methods For Intervertebral Body Fusion Devices) and a load-induced subsidence static test was performed according to the ASTM F2267-04 standard (Standard Test Method for Measuring Load Induced Subsidence of Intervertebral Body Fusion Device Under Static Axial Compression). 

#### 3.2.1. Static Compression Test

The static compression test was performed using a static material testing machine (MTS Insight 100) with software for data acquisition (TestWorks 4 Advanced Product Package), equipped with an MTS 569330-01 load sensor and a MESSPHYSIK ME46 videoextensometer, integrated with TestWorks. During the tests, three samples of each type of intervertebral cages were used. The samples were gamma-sterilized with the maximum permissible dose of 40 kGy. The samples were mounted in a special fixture, where the testing machine actuator was connected to the load axis by a universal joint, according to ASTM F2077-11. The compression tests were performed with the use of steel blocks, under displacement control, at a rate of 1.3 mm/minute, according to ASTM D695-10. The load versus displacement ratio was recorded until the occurrence of implant failure or reaching ofa 1 mm deformation. Failure was defined as a plastic deformation or sample breakage.

#### 3.2.2. Load Induced Subsidence Static Test

The test was performed according to ASTM F2267-04 standards. Compression tests were performed under displacement control, at a rate of 6 mm/minute. Load versus displacement was recorded until reaching a 3 mm displacement. The force value needed for 3-mm subsidence of the implant into the test blocks made of Grade 15 polyurethane foam was determined.

### 3.3. Wettability Test

Wettability of the samples was assessed by measurement of water contact angle using the DSA 30 Kruss goniometer. Water contact angle (WCA) was evaluated by the static contact angle method (sessile drop technique), using ultrapure water obtained from Milli-Q^®^ Water Purification System (Merck, Warsaw, Poland). The wetting behavior of the samples was determined by averaging the mean contact angles obtained with at least 12 measurements performed for 3 independent samples.

### 3.4. Bioactivity Test

The bioactivity assessment was conducted in accordance with ISO 23317:2012 procedure. In brief, the materials were put in polypropylene falcon conical tubes and soaked in a simulated body fluid (SBF) at 37 °C for 28 days. SBF (pH 7.4) is an inorganic solution having similar ion concentrations to those of human blood plasma. After 28 days of incubation, materials were taken out from the SBF and softly rinsed with deionized water, and dried in a desiccator. Then, apatite crystals that formed on the surfaces of the materials were examined using a scanning electron microscope (SEM; JEOL JCM-6000Plus, Tokyo, Japan). For this purpose, the samples were coated with a thin gold layer (10 nm) under a high vacuum, using a sputter coater. SEM images were obtained in an accelerating voltage of 5 kV and 15 kV.

Additionally, the specimens were decalcified using 0.5 M HCl (Avantor Performance Materials, Gliwice, Poland) to determine the concentration of deposited calcium phosphates on the surfaces of the materials. Ion concentrations were evaluated using a calcium and phosphorus detection kit, respectively (BioMaxima, Lublin, Poland).

### 3.5. Cell Culture Tests

#### 3.5.1. Cell Culture

Cell viability, adhesion, and growth assessments were conducted using osteoblast cell lines: normal human fetal osteoblast cell line (hFOB 1.19, ATCC-LGC Standards, Teddington, UK) and normal mouse calvarial preosteoblast cell line (MC3T3-E1 Subclone 4, ATCC-LGC Standards, Teddington, UK), which were cultured as described earlier [18]. In brief, the hFOB 1.19 cells were cultured in a 1:1 mixture of DMEM/Ham’s F12 medium without phenol red, supplemented with fetal bovine serum, penicillin-streptomycin, and G418 (Sigma-Aldrich Chemicals, Warsaw, Poland) and maintained at 34 °C (95% air, 5% CO_2_). In turn, the MC3T3-E1 cells were cultured in an alpha MEM medium (Gibco, Life Technologies, Carlsbad, CA, USA), supplemented with fetal bovine serum, penicillin-streptomycin (Sigma-Aldrich Chemicals, Warsaw, Poland) and maintained at 37 °C (95% air, 5% CO_2_). Evaluation of the effect of biomaterials on osteogenic differentiation was carried out using hFOB 1.19 cells and human bone marrow-derived mesenchymal stem cells (BMDSCs) (ATCC-LGC Standards, Teddington, UK). The BMDSCs cells were maintained as described previously [47]. Briefly, the BMDSCs were cultured in Mesenchymal Stem Cell Basal Medium (ATCC-LGC Standards, Teddington, UK) supplemented with the Bone Marrow-Mesenchymal Stem Cell Growth Kit (ATCC-LGC Standards, Teddington, UK) and penicillin-streptomycin, and maintained at 37 °C (95% air, 5% CO_2_). Before all cell culture tests, discs of the materials were put in the wells of a 48-multiwell plate and preincubated overnight in an appropriate complete growth medium at 37 °C.

#### 3.5.2. Cell Viability Assessment

The hFOB 1.19 and MC3T3-E1 cells were seeded directly on the materials placed in the wells of a 48-multiwell plate in 500 mL of growth medium at a concentration of 5 × 10^4^ cells per sample. After 48 h of culture, osteoblasts cultured on the surface of the materials were stained using the Live/Dead Double Staining Kit (Sigma-Aldrich Chemicals, Warsaw, Poland), according to the manufacturer’s procedure, and visualized by means of a confocal laser scanning microscope (CLSM, Olympus Fluoview equipped with FV1000, Olympus Polska Sp. z o. o., Warsaw, Poland).

#### 3.5.3. Cell Adhesion and Morphology Assessment

To assess adhesion and morphology of hFOB 1.19 and MC3T3-E1 cells, osteoblasts were seeded directly on materials placed in wells of a 48-multiwell plate in 500 mL of growth medium at a concentration of 2 × 10^4^ cells per sample and cultured for 48 h. Then, cells cultured on the surface of the materials were subjected to fixation and permeabilization by using 3.7% paraformaldehyde and 0.2% Triton X-100 (Sigma-Aldrich Chemicals, Warsaw, Poland), respectively. Then, the samples were blocked with 1% bovine serum albumin (Sigma-Aldrich Chemicals, Warsaw, Poland) for 30 min. Materials seeded with mouse preosteoblasts (MC3T3-E1 cells) were incubated overnight at 4 °C with primary mouse-specific anti-vinculin rabbit antibody (Abcam, Cambridge, UK). Subsequently, the materials were washed with a physiological buffer solution and incubated for 1 h at room temperature with secondary Alexa-Fluor^®^488 goat anti-rabbit IgG antibody (Abcam, Cambridge, UK). All samples (seeded with mouse and human cells) were stained using AlexaFluor635-conjugated phallotoxin (Invitrogen, Carlsbad, California, USA) and DAPI (Sigma-Aldrich Chemicals, Warsaw, Poland) to show cytoskeleton filaments and nuclei, respectively. Stained cells were visualized by CLSM. 

#### 3.5.4. Cell Proliferation Assessment

The hFOB 1.19 and MC3T3-E1 cells were seeded directly on the materials placed in wells of 48-multiwell plates in 500 mL of growth medium at a concentration of 5 × 10^3^ cells per sample. After 3, 48, and 96 h of culture, the number of cells was determined by using colorimetric assay: Cell Counting Kit-8 (Sigma-Aldrich Chemicals, Warsaw, Poland), performance of the assay was in accordance with the manufacturer’s procedure. Additionally, after 96 h of culture, visualization of osteoblasts on the surface of the materials was carried out using CLSM and scanning electron microscopy (SEM). Before visualization, the samples were fixed, as described in Section 3.5.3. For CLSM analysis, the samples were stained with AlexaFluor635-conjugated phallotoxin and DAPI. For SEM visualization, the samples were dehydrated in graded ethanol concentrations (from 35% to 99.8%), as was described previously [3]. Then, the materials were coated with a thin gold layer (10 nm) and subjected to visualization, using SEM (accelerating voltage of 5 kV was applied).

#### 3.5.5. Evaluation of Osteogenic Differentiation

The hFOB 1.19 and BMDSC cells were seeded directly on the materials placed in the wells of 48-multiwell plates in 500 mL of growth medium at a concentration of 5 × 10^4^ cells per sample and cultured at 37 °C. After 24-h culture, the growth medium was exchanged for osteogenic medium, containing 10 mM β-glycerophosphate, 50 μg/mL ascorbic acid, and 10^−7^ M dexamethasone (Sigma-Aldrich Chemicals, Warsaw, Poland). The cells were maintained in the osteogenic medium for 21 days. Every 3rd day, half of the osteogenic medium was replaced with a fresh portion. On the 7th and 14th day of the experiment, osteogenic markers (type I collagen—Col I, bone sialoprotein—BSP, osteocalcin—OC) were determined in cell lysates using appropriate ELISA kits (EIAab ELISA kit, Wuhan, China). The cell lysates were prepared by two freeze–thaw cycles and then underwent sonication at 30% amplitude for 30 s, as described earlier [43]. ELISA results were normalized to the total cellular proteins assessed by the BCA Protein Assay Kit (ThermoFisher Scientific, Waltham, Massachusetts, USA), and expressed as ng of the osteogenic marker per mg of total cellular proteins. 

ECM mineralization in response to tested samples was evaluated by quantitative mineral determination based on alizarin red S staining (ARS) and by IF staining of Col I fibers, acting as a framework for mineral deposition. Before experiments, the samples were fixed, as described in Section 3.5.3. Quantitative mineral determination based on ARS staining was performed according to the procedure described previously [44]. Briefly, cells grown on the samples were stained with ARS, calcium bonding dye (Sigma-Aldrich Chemicals, Warsaw, Poland). Stained cells were then treated with 10% acetic acid (Sigma-Aldrich Chemicals) to dissolve mineral-ARS complex, subjected to heating for 10 min at 85 °C and the resultant solutions were neutralized with 10% ammonium hydroxide (Avantor Performance Materials, Gliwice, Poland). The absorbance of the solutions was measured at 405 nm. The accurate amounts of mineral deposition were estimated from the calibration curve made for known concentrations of calcium phosphate. For IF, fixed samples were incubated overnight at 4 °C with primary human-specific goat anti-collagen I antibody (Col1a1/Col1a2) (Abnova, Taoyuan City, Taiwan). Subsequently, the samples were washed with a physiological buffer solution and incubated for 1 h at room temperature with secondary AlexaFluor^®^647 donkey anti-goat IgG antibody (Abcam, Cambridge, UK) and DAPI. Stained cells were visualized by CLSM. 

### 3.6. Statistical Analysis

Statistical analyses were performed using GraphPad Prism 8.0.0 Software (GraphPad Software Inc., California, CA, USA). All experiments were conducted at least in triplicate. Unpaired t-test, or One-way ANOVA followed by Tukey’s multiple comparison test, was used to estimate statistical differences (*p* < 0.05) between samples.

## 4. Conclusions

The conducted research demonstrated that application of SLM for the production of a mesh Ti6Al4V intervertebral cage allows the obtaining of a metallic implant with improved mechanical behavior, compared to the commonly used PEEK cage. Mechanical tests revealed that the mesh Ti6Al4V implant has significantly higher compressive strength and a significantly lower tendency to subside, compared to the PEEK implant. Moreover, both Ti6Al4V samples (solid and mesh) are characterized by superior bioactivity and osteoconductivity, compared to the PEEK material. Osteogenic differentiation of osteoblasts and mesenchymal stem cells was similar on all tested samples. However, 3D-printed titanium samples have the ability to significantly intensify the mineralization process. Importantly, introduction of mesh porosity to the 3D-printed Ti6Al4V sample slightly affects its bioactivity and biocompatibility, but mesh titanium samples still have superior biological properties, compared to the PEEK material. Based on the research conducted, it may be concluded that the 3D-printed mesh Ti6Al4V intervertebral cage possesses all the features of the optimal spinal implant, since it carries low risk of implant subsidence and provides good osseointegration at the bone-implant interface.

## Data Availability

The raw/processed data required to reproduce these findings can be obtained from the corresponding author (agata.przekora@umlub.pl) upon reasonable request.
